# Micro-rheological properties of lung homogenates correlate with infection severity in a mouse model of *Pseudomonas aeruginosa* lung infection

**DOI:** 10.1038/s41598-020-73459-5

**Published:** 2020-10-05

**Authors:** Xabier Murgia, Andreas M. Kany, Christian Herr, Duy-Khiet Ho, Chiara De Rossi, Robert Bals, Claus-Michael Lehr, Anna K. H. Hirsch, Rolf W. Hartmann, Martin Empting, Teresa Röhrig

**Affiliations:** 1 Department of Drug Delivery (DDEL), Helmholtz-Institute for Pharmaceutical Research Saarland (HIPS)-Helmholtz Centre for Infection Research (HZI), Helmholtz Institute for Pharmaceutical Research Saarland, Campus E8.1, 66123 Saarbrücken, Germany; 2 Department of Drug Design and Optimization (DDOP), Helmholtz-Institute for Pharmaceutical Research Saarland (HIPS)-Helmholtz Centre for Infection Research (HZI), Campus E8.1, 66123 Saarbrücken, Germany; 3 German Centre for Infection Research (DZIF), Partner Site Hannover-Braunschweig, Saarbrücken, Germany; 4 Department of Internal Medicine V – Pulmonology, Allergology, Critical Care Medicine, Saarland University Hospital, 66421 Homburg, Germany; 5 Department of Pharmacy, Saarland University, Campus E8.1, 66123 Saarbrücken, Germany; 6 Present Address: Kusudama Therapeutics, Parque Científico y Tecnológico de Gipuzkoa, 20014 Donostia-San Sebastián, Spain

**Keywords:** Biomarkers, Drug development, Infection

## Abstract

Lung infections caused by *Pseudomonas aeruginosa* pose a serious threat to patients suffering from, among others, cystic fibrosis, chronic obstructive pulmonary disease, or bronchiectasis, often leading to life-threatening complications. The establishment of a chronic infection is substantially related to communication between bacteria via quorum-sensing networks. In this study, we aimed to assess the role of quorum-sensing signaling molecules of the *Pseudomonas* quinolone signal (PQS) and to investigate the viscoelastic properties of lung tissue homogenates of PA-infected mice in a prolonged acute murine infection model. Therefore, a murine infection model was successfully established via intra-tracheal infection with alginate-supplemented *Pseudomonas aeruginosa* NH57388A. Rheological properties of lung homogenates were analyzed with multiple particle tracking (MPT) and quorum-sensing molecules were quantified with LC–MS/MS. Statistical analysis of bacterial load and quorum-sensing molecules showed a strong correlation between these biomarkers in infected lungs. This was accompanied by noticeable changes in the consistency of lung homogenates with increasing infection severity. Furthermore, viscoelastic properties of the lung homogenates strongly correlated with bacterial load and quorum sensing molecules. Considering the strong correlation between the viscoelasticity of lung homogenates and the aforementioned biomarkers, the viscoelastic properties of infected lungs might serve as reliable new biomarker for the evaluation of the severity of *P. aeruginosa* infections in murine models.

## Introduction

Carbapenem-resistant *Pseudomonas aeruginosa* is one of the most critical antibiotic-resistant bacteria as recently classified by the World Health Organization^1^, which is the reason why current anti-infective research is dedicated to the development of new treatment options against this pathogen^2,3^. Infections with *P. aeruginosa* lead to severe complications in chronic lung diseases like cystic fibrosis (CF), chronic obstructive pulmonary disease (COPD) and non-CF bronchiectasis (NCFB), often leading to significantly increased morbidity and mortality^4,5^. Especially in CF, abnormal mucus morphology, characterized by increased thickness and volume, reduces mucociliary clearance, providing a favorable environment for bacterial colonization^6^. Despite antibiotic treatment, these infections can become persistent, causing recurring exacerbations and providing a high possibility for antibiotic resistance development^4,5^. In early stages of infection, *P. aeruginosa* is able to form impenetrable biofilms and in later stages of infection may further mutate to a mucoid phenotype, worsening the overall disease prognosis^5^. In order to establish and maintain host infections, *P. aeruginosa* upregulates the expression of several virulence factors including biofilm and additional alginate production via quorum-sensing (QS) systems. *P. aeruginosa*-specific transcriptional multi-virulence factor regulator (mvfR), also known as pqsR, is activated by the alkylquinolone PQS (2-heptyl-3-hydroxy-4(1*H*)-quinolone) and its biosynthetic precursor HHQ (2-heptyl-4-quinolone). As one of at least four QS systems in *P. aeruginosa*, pqsR mediates pyocyanin and hydrogen cyanide production as well as biofilm formation and alginate production in particular^7,8^. In CF diagnostics, the PQS precursor HHQ (2-heptyl-4(1*H*)-quinolone) can even be used to identify current *P. aeruginosa* infections from patients’ plasma samples^9^.

In order to study *P. aeruginosa* infections in the context of chronic lung diseases, the use of animal models has become indispensable. Nevertheless, developing such chronically-infected, yet reproducible models with reliable readouts constitutes a great experimental challenge with just a few successful examples reported in the literature^10,11^. Facchini et al*.* utilized the non-mucoid clinical *P. aeruginosa* strain RP73 embedded in agar beads to chronically infect mice, while Hoffmann et al*.* used the mucoid strain NH57388A in alginate suspension also in mice. Common readouts for the infection status of these in vivo infection models embrace colony-forming unit (CFU) count, QS signal molecules, histopathological analysis, and immunological biomarkers. Additionally, Hoffmann et al*.* determined the production of endogenously produced bacterial alginate in lung homogenates. Bacterial alginate production and mucus hypersecretion are two well-known factors affecting the viscoelastic properties of the pulmonary lining fluid^12^.

Taking inspiration from the aforementioned models, our aim was to set-up an in vivo model of prolonged acute *P. aeruginosa* infection in order to study the role of pqsR and its signal molecules in lung infection. We opted for a sub-chronic model with a 72-h end point employing *P. aeruginosa* isolate NH57388A strain in alginate suspension as the inoculum applied via the intratracheal instillation (i.t.) route. In the early stages of model establishment, it became macroscopically apparent that lung homogenates generated from severely infected mice had a higher viscoelasticity compared to those corresponding to less severely infected animals. This observation prompted us to postulate that mechanical properties of lung homogenates in this model could correlate with the biomarker levels of infection severity. We therefore investigated the micro-rheological characteristics of lung homogenates by means of multiple particle tracking (MPT). This technique consists in tracking the Brownian motion of tracer particles dispersed within a complex biological fluid and allows determining the mechanical properties of the material by measuring the displacement of the tracer particles at different time scales^13,14^. To test our hypothesis, we investigated the correlation between CFU counts and the amount of pqsR molecules (PQS and HHQ) determined in the lung homogenates of mice infected with *P. aeruginosa NH57388A* with the mechanical properties of the same lung homogenates measured by means of MPT micro-rheology.

## Materials and methods

### Bacterial culture

*Pseudomonas aeruginosa* strain NH57388A was grown in lysogeny broth (LB) medium at 37 °C, overnight, 170 rpm and adjusted to an OD_600_ = 1, and diluted 1:1 with sodium alginate solution (in 0.9% sterile NaCl, final concentration 11 mg/mL) to yield the desired bacterial density required for infection.

### Mouse experiment

Mouse experiments were approved based on ethical considerations by the animal welfare committee of the ‘Landesamt für Soziales, Gesundheit und Verbraucherschutz’ of the State of Saarland in accordance with the national guidelines for animal treatment on August, 17th, 2017 (22/2017). In two different experiments, ten-week old female C57BL/6 N mice were slightly anesthetized intraperitoneally with 2.025 mg of ketamine hydrochloride (Ursotamin, Serumwerk Bernburg, Germany) and 0.12 mg of xylazine hydrochloride (Xylazin, WDF, Serumwerk Bernburg) per mouse. Lidocaine hydrochloride (Xylocain; Aspen Pharma, Irland) was used for local anesthesia of the epiglottis before endotracheal intubation with a flexible cannula and infection with 40 µL viable *P. aeruginosa* NH57388A culture. Bacterial load was determined to be 5 × 10^7^ CFU by plating serial dilutions on LB-agar plates. During the course of 72 h after infection, a high weight loss was observed (mean 20 ± 1.5%) at a survival rate of 83%, whilst the overall appearance of the animals stayed within the predefined scores for animal distress. 24 h (N = 1), 48 h (N = 1), and 72 h (N = 10) after infection, mice were euthanized, the lungs removed and immediately stored on ice. They were then homogenized with an ultra turrax homogenizer (IKA, Staufen im Breisgau, Germany) at 20,000 rpm in 500 µL ice-cold sterile phosphate buffered saline and immediately stored at − 80 °C until analysis.

### LC–MS/MS analysis of alkylquinolones

Alkylquinolone signal molecules PQS (2-heptyl-3-hydroxy-4-quinolone), HHQ (2-heptyl-4-quinolone), HQNO (N-oxo-2-heptyl-4-Hydroxyquinoline) in murine lung homogenates were analyzed via LC–MS/MS. Stable isotope standards (d4HHQ and d4PQS, 200 pmol each) were added to lung homogenates prior to extraction with 1 mL ethyl acetate. After extraction under vigorous shaking in an orbital shaker (10 min, 1750 rpm, IKA, Staufen, Germany) 800 µL supernatant was dried and reconstituted with 400 µL methanol. Lower limits of quantification were determined to be 0.25 pmol/lung for HHQ and HQNO, 2.5 pmol/lung for PQS. LC–ESI–MS/MS conditions were as follows: Dionex Ultimate 3000 HPLC coupled to triple quad mass spectrometer TSQ Quantum Access Max (Thermo Fisher Scientific, Waltham, MA, USA), Zorbax Eclipse XDB 80 Å C18 5 µm 4.6 × 50 mm equipped with guard column (Agilent, Santa Clara, CA, USA); eluent A—H_2_O with 0.1% trifluoroacetic acid (TFA), 0.1% pentafluoropropionic acid (PFPA), 0.1% heptafluorobutyric acid (HFBA); eluent B—acetonitrile with 0.1% TFA, 0.1% PFPA, 0.1% HFBA; isocratic 50% A; flow—0.7 mL/min. Instrument parameters: Spray voltage 3500 V, vaporizer temperature 370 °C, sheath gas pressure 35 psi, aux gas pressure 30 psi, capillary temperature 270 °C, collision pressure 1.5 mTorr, positive ionization mode. For QS analyte reaction monitoring parameters please see Table S1.

### Multiple particle tracking

Red-fluorescent, carboxyl-coated polystyrene tracer particles with a nominal size of 200 nm were purchased from Invitrogen. The surface of the particles was modified by coating them with a dense layer of polyethylene glycol (PEG, 5000 Da), as previously described^15^. Briefly, a 0.2% (*w/w*) particle suspension was diluted eightfold in ultrapure water. An excess of 4-(4,6-dimethoxy-1,3,5-triazin-2-yl)-4-methyl-morpholinium chloride (DMTMM) was added to the particle suspension. Following 3 h-activating reaction, an excess amount of methoxy-PEG-amine, prepared in ultrapure water at 4% (*w/w*), was added. The resulting solution was carried out at room temperature for 24 h. The particle suspension was then centrifuged and washed 3 times with ultrapure water. The particles were re-suspended in ultrapure water to a 0.2% (*w/w*) concentration. PEG-coated particle suspensions were characterized in terms of particle diameter and surface charge by nanoparticle tracking analysis (NTA, Nanosight, Malvern Instruments) and dynamic light scattering (Z-sizer Nano ZSP, Malvern Instruments), respectively. Mean hydrodynamic particle size slightly increased after PEGylation from 192 ± 28 nm to 218 ± 48 nm (mean ± standard deviation). The surface charge was determined in 1 mM NaCl solution and showed an increase after PEGylation from − 24 ± 0.83 mV to neutral charge (− 0.2 ± 1.45 mV) indicative of successful surface PEG coating of the particles.

Lung homogenates were thawed gradually and allowed to reach room temperature. 30 µL of each lung homogenate sample were mixed with 1 µL of the tracer particle stock (0.1% w/v). The whole mixture was transferred to a chamber mounted onto a microscope slide (Gene Frame, Thermo Fisher Scientific) and sealed with a cover slip. Samples were incubated for at least one hour before imaging. Video sequences were captured with a confocal laser scanning microscope (Leica TCS SP 8; Leica, Mannheim, Germany) equipped with a DPSS 561 laser and a HC PL APO CS 63 × objective (NA of 1.2). Experiments were performed at 23 °C. For each sample two independent fields were recorded at a resolution of 0.142 µm per pixel and a frame rate of 28 frames per second. The physical length of each microscopic field represents an area of 147 × 37 µm. Image processing and analysis were performed as described by Ho et al.^15^.

### Statistical analysis

**Figure 1:**
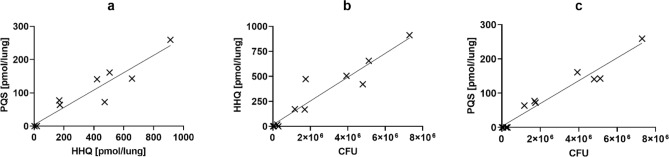
Dependence of signal molecule production on bacterial load. (**a**) Scatterplot and linear regression of the quorum sensing signaling molecules PQS (2-heptyl-3-hydroxy-4(1H)-quinolone) *vs* HHQ (2-heptyl-4-quinolone) detected in murine *Pseudomonas aeruginosa* NH57388A lung infection samples. (**b**,**c**) Scatterplot and linear regression of PQS (B) and HHQ (C) *vs* corresponding colony forming units (CFU). See also Fig. S1.

**Figure 2:**
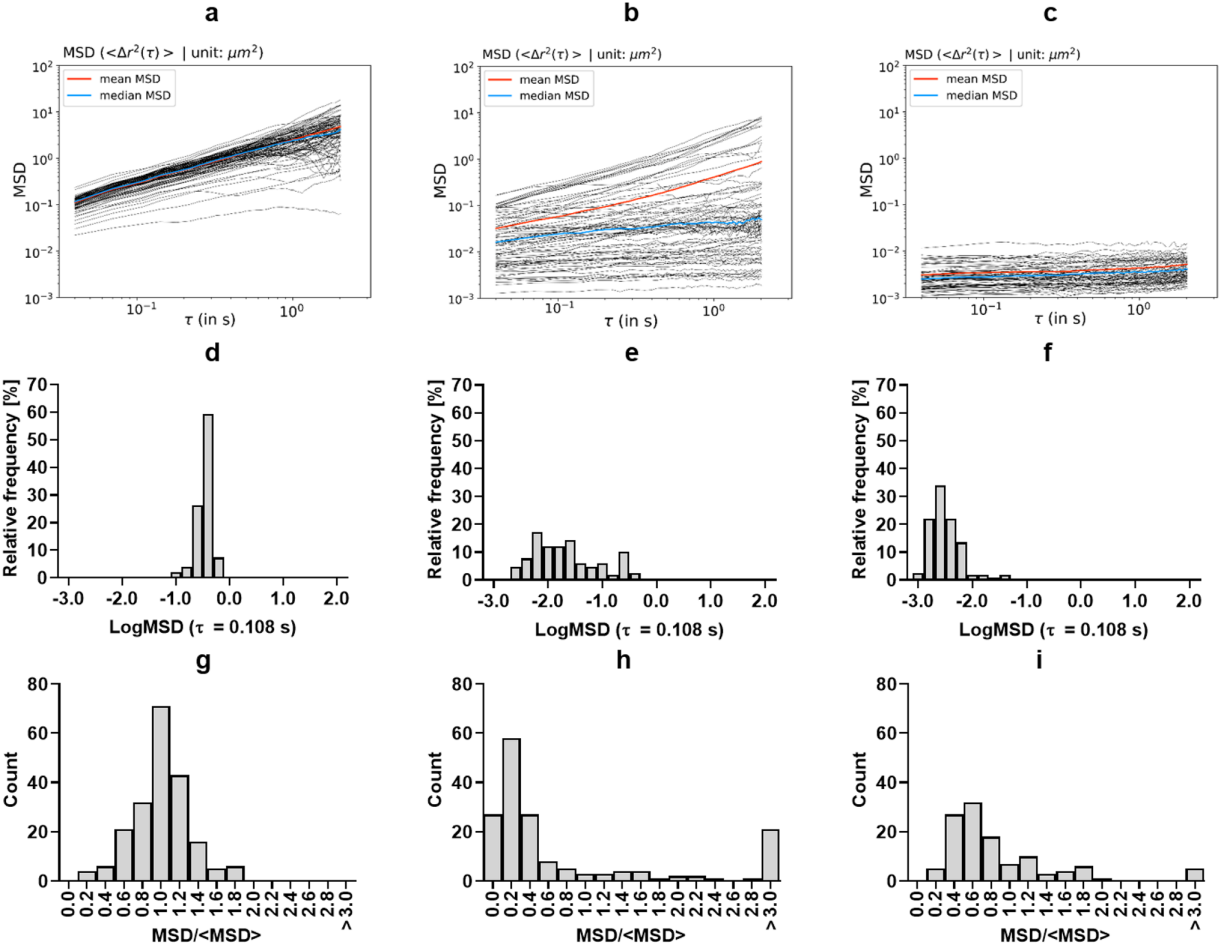
Representative mean squared displacement (MSD) plots as a function of the time scale (*τ*) of polyethylene glycol-coated polystyrene particles. (**a**–**c**) mean squared displacement (MSD) of each individual tracer particle trajectory at different *τ* in representative lung homogenates of murine *Pseudomonas aeruginosa* NH57388A lung infection samples. All animals were infected with the same bacterial inoculum but developed different degrees of infection that correlated with low (**a**), medium (**b**), and high (**c**) viscoelasticity. The mean and the median of all individual particles are indicated by the red and blue lines, respectively. (**d**–**f**) corresponding distribution of the logMSD at a *τ* = 0.1 s*.* See also Fig. S2. Tracer particles had a mean diameter of 218 nm. (**g**–**i**) Representative mean squared displacement (MSD) distributions at *τ* = 0.108 s normalized by the ensemble-average MSD (〈MSD) at *τ* = 0.108 s. See also Fig. S3.

**Figure 3:**
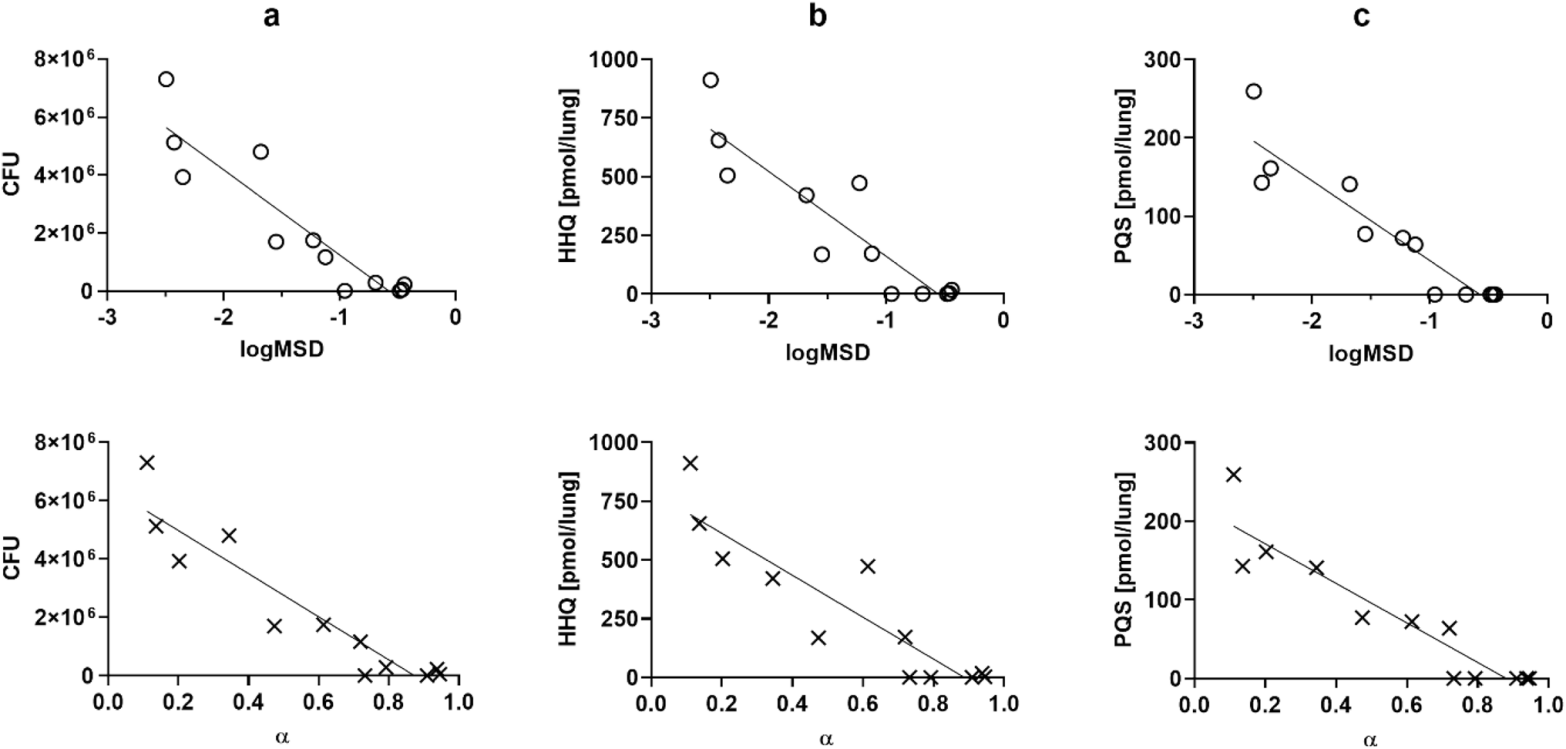
Rheological properties strongly correlate with bacterial biomarkers. Scatterplot and linear regression of colony forming units (CFU; **a**), PQS (**b**) and HHQ (**c**) *vs* tracer particle logMSD (logarithm of mean square displacement at *τ* = 0.1 s) and exponential coefficient α. See also Fig. S4.

Regression curves shown in Figs. 1, 3, Figs. S1, S4 and the corresponding Pearson’s correlation coefficients (r, Table S2 and S3) were determined using the simple linear regression function of GraphPad Prism 8.4.2 (GraphPad Software, San Diego, CA, USA). Histograms in Figs. 2, Figs. S2 and S3 were created using the frequency distribution function of Prism. The presented coefficients were determined from 12 individual samples, which can be separated into two independent experiments with 4 and 8 individual samples, respectively. Separate values for r representing correlation in the individual experiment as shown in Figs. S1 and S4 were determined using the same procedure. Individual histograms are presented in Figs. S2 and S3.

## Results and discussion

### Bacterial load of mouse lungs strongly correlates with signal-molecule levels

In order to study chronic *P. aeruginosa* infections of the lung and the role of the pqsR quorum sensing system, a chronic murine lung infection model was established in accordance with Hoffmann et al.^11^. In the present study, infection in mice was accomplished via an alginate-based intra-tracheal inoculation with mucoid *P. aeruginosa* NH57388A. Upon removal of the murine lungs 72 h post infection, heterogeneously distributed lesions of the lung tissue were macroscopically visible. Throughout homogenization and sample-extraction procedures we noticed different consistencies of lung homogenates for individual animals. In significant cases, lung homogenates were not of a fluid consistency and showed an apparent higher viscosity and an elastic behavior compared to other samples.

The median CFU count in extracted organs was 1.4 × 10^6^ CFU/lung, showing a wide range across the animals (range = 7.4 × 10^3^–7.3 × 10^6^), which indicates a high variance of infection status in individual mice. In regard to the inoculum, the CFU count shows a high clearance of bacteria during 72 h post infection while a severe infection status is maintained in most cases based on weight loss and animal behavior. The individual variability observed in terms of CFU count was mirrored by the QS signal molecules quantified in lung homogenates. HHQ and PQS correlated strongly (r = 0.9548, Fig. 1a), as was expected. Since QS is active in high cell densities, we anticipated further that more signal molecules could be found in those lung homogenates with the highest CFU counts. Indeed, Fig. 1b,c shows a positive correlation of CFU counts with HHQ (r = 0.9523) and PQS (r = 0.9769), confirming this hypothesis and validating the infection model. To the best of our knowledge, this is the first time that a very strong correlation between PQS or HHQ and bacterial load in biological samples has been demonstrated. In two studies by Barr and coworkers^9,16^, the correlation of HHQ concentration in plasma samples from CF patients with bacterial load of sputum was significant in a moderate to weak range (r = 0.28 and r = 0.51) and in a study by Collier and coworkers, a correlation between PQS and bacterial density in sputum samples from CF patients was shown but not quantitatively^17^. Considering the mucoid feature of the *P. aeruginosa NH57388A* strain, we investigated whether the determined bacterial load and the detected signal molecule levels could further be correlated with the mechanical properties of the lung homogenate.

### Micro-rheology of tissue homogenates serves as reliable biomarker

A volume of 30 µL-aliquots of individual lung homogenates were mixed with red-fluorescent, 200 nm particles coated with a dense layer of polyethylene glycol (PEGylation), and their Brownian motion was monitored by fluorescence video microscopy. Particle PEGylation is a well-established nanoparticle coating strategy that reduces the interaction between particles and biological entities^18,19^. Therefore, it is assumed that such particles are bio-inert and that those particles, which appear immobile in video microscopy are sterically hindered rather than chemically adsorbed. The mean squared displacement (MSD or 〈Δ*r*^*2*^) of the tracer particles at each time scale (*τ*) was determined from video microscopy images by computing the x–y coordinates of each particle in each video frame using a custom-written *Python* script, as previously described^15,20^:

$$ \langle \Delta r^{2} \left( \tau \right)\rangle = \left\{ {x\left( {t + \tau } \right) - x\left( t \right)} \right\}^{2} + \left\{ {y\left( {t + \tau } \right) - y\left( t \right)} \right\}^{2} $$

Figure 2a–c depicts MSD plots of representative lung tissue homogenates with different macroscopic consistencies (low, medium and high apparent viscoelasticty, respectively). Figure 2d–f describes the logMSD distribution of these samples at a τ of 0.108 s. Particles with a diffusive behavior increase their MSD as a function of the *τ* (Fig. 2a, video S1), whereas those particles trapped by the biomacromolecular matrix of the lung homogenate show a constant MSD at any *τ* (Fig. 2c, video S2)*.* Lung homogenates simultaneously displaying viscous and elastic areas show MSDs corresponding to diffusive and hindered particle trajectories (Fig. 2b, video S3). From the log–log MSD plots*,* the slope (*α*) of each particle trajectory can be calculated:

$$ \alpha = \frac{{{\text{d}}\log \langle \Delta r^{2} \rangle \left( \tau \right)}}{{{\text{d}}\log \left( \tau \right)}} $$

The slope describes the diffusive properties of the tracer particles. For particles diffusing through a Newtonian fluid *α* = 1. Conversely, for particles immobilized by an elastic environment *α* = 0^21^.

The wide variability observed for the CFU counts and QS signal molecules was also evidenced by the micro-rheology study of the lung homogenates. The displacement of the tracer particles as well as the slope of the MSD trajectories varied significantly across the 12 independent lung samples analyzed in this study. The range of the mean logMSD (*τ* = 0.108 s) expanded from − 0.44 to − 2.49 corresponding to samples with the most diffusive and immobile particles, respectively (Fig. S2). Similarly, the mean α at a *τ* = 0.108 s ranged between 0.94, for the sample with the most diffusive particles, and 0.11 for the most elastic sample. This wide range of MSD and *α* across individual samples implies different cross-linking densities between the constituents of the lung homogenates of different animals. Such differences clearly point to a different biochemical composition of the independent lung homogenates. In those lung homogenates corresponding to the most severe infection status (i.e. higher CFU), tracer particles appear almost exclusively immobilized, which indicates the existence of an interconnected biomacromolecular network with a pore size below the diameter of the tracer particles. Additionally, micro-scale inhomogeneity was detected in individual samples, which simultaneously showed diffusive and immobile particles. To quantify the level of inhomogeneity, the MSD distributions at *τ* = 0.108 s normalized by the ensemble-average MSD (〈MSD at *τ* = 0.108 s) were plotted^13^. Figure 2 g–i shows three representative MSD/〈MSD distribution plots corresponding to the same examples as in Fig. 2a–f:

For the sample with an apparent low viscoelasticity, the distribution is symmetric about the mean, corresponding to a homogeneous fluid. Conversely, for the samples with apparent intermediate and high viscoelasticity no symmetry can be observed and most trajectories appear to the left side of the mean, indicative of sample inhomogeneities at the micro-scale where diffusive, sub-diffusive and immobile particle trajectories can be observed.

The diffusion coefficient (D) was calculated for particle trajectories with an *α* > 0.8:

$$ \langle \Delta r^{2} \left( \tau \right)\rangle = 4{\text{D}}\tau $$

which was further used to calculate the viscosity (η) of the material surrounding the tracer particles by applying the Stokes–Einstein relation:

$$ \eta = \frac{{k_{B} T}}{3d\pi D} $$

where *d* is the particle diameter, *K*_*B*_ the Boltzmann constant and *T* the temperature.

The thermal motion of non-interacting immobile particles is determined by the elastic properties of the surrounding network. Therefore, for particle trajectories with an *α* < 0.2 the so-called plateau modulus G_0_ of this network was estimated using the following expression^22^:

$$ G_{0} = \frac{{2K_{B} T}}{{3{\text{r}}\pi \langle \Delta r^{2} \rangle }} $$

where 〈$\langle \Delta r^{2} \rangle$ is the time independent average MSD and *r* is the radius of the tracer particles.

According to the classical theory of rubber elasticity, the modulus G_o_ can be related to the mesh size ξ of the elastic network characterizing the average distance between adjacent crosslinks in the network^23^.

$$ \xi = (K_{B} T/G_{0} )^{1/3} $$

The results from the MPT analysis are summarized in Table 1.

**Table 1:** Summary of multiple particle tracking (MPT) analysis of each lung homogenate from 12 independent mice.

	〈MSD *τ* = 0.108 s	D *τ* = 0.108 s	α	η	G_0_	*ξ*
	[µm^2^]	[µm^2^/s]		[mPa*s]	[Pa]	[nm]
Mouse #1	0.368 ± 0.12	0.901 ± 0.26	0.94 ± 0.11	2.41 ± 0.75	^a^	^a^
Mouse #2	0.376 ± 0.10	0.891 ± 0.22	0.93 ± 0.11	2.41 ± 0.88	^a^	^a^
Mouse #3	0.348 ± 0.10	0.828 ± 0.21	0.91 + 0.12	2.60 ± 0.85	^a^	^a^
Mouse #4	0.228 ± 0.16	0.764 ± 0.23	0.73 ± 0.34	3.03 ± 1.99	1.57 ± 1.07	157 ± 52
Mouse #5	0.161 ± 0.17	0.794 ± 0.29	0.61 ± 0.31	3.05 ± 1.9	0.75 ± 0.59	208 ± 72
Mouse #6	0.277 ± 0.16	0.746 ± 0.35	0.79 ± 0.22	3.32 ± 1.58	^a^	^a^
Mouse #7	0.182 ± 0.16	0.673 ± 0.33	0.72 ± 0.32	4.23 ± 3.60	1.56 ± 0.90	146 ± 24
Mouse #8	0.057 ± 0.09	0.520 ± 0.22	0.34 ± 0.31	5.03 ± 3.07	1.16 ± 1.01	179 ± 59
Mouse #9	0.086 ± 0.11	0.512 ± 0.24	0.47 ± 0.35	6.22 ± 6.03	1.75 ± 1.40	160 ± 54
Mouse #10	0.004 ± 0.005	^b^	0.11 ± 0.10	^b^	3.03 ± 1.51	118 ± 24
Mouse #11	0.006 ± 0.005	^b^	0.20 ± 0.17	^b^	2.84 ± 1.67	123 ± 27
Mouse #12	0.006 ± 0.010	^b^	0.13 ± 0.12	^b^	2.48 ± 1.21	129 ± 39

〈MSD, ensemble-averaged mean squared displacement from all trajectories; *D* diffusion; *α* exponential coefficient; *η* viscosity; *G*_*o*_ plateau modulus; *ξ* mess size. D and η were average from all trajectories with α > 0.8, whereas G_o_ and ξ were averaged from trajectories with α < 0.2.

^a^Not enough trajectories (less than 10) with α < 0.2 to compute G_0_ and ξ.

^b^Not enough trajectories (less than 10) with α > 0.8 to compute D and η. Mean ± Standard deviation are shown.

In three lung homogenates out of twelve infected mice samples, there were not enough diffusive particles (*α* > 0.8) to compute D or η. These samples displayed the lowest mean *α* values (0.11–0.20), the highest G_0_ (2.48–3.03 Pa), and a pore size below 130 nm. The η of the lung homogenate fluid surrounding the tracer particles could be determined from nine out of twelve infected mice samples and it ranged between 2.41 and 6.22 mPa*s, representing two- and sixfold the viscosity of water at 20 °C (1 mPa*s).

Remarkably, the mean logMSD and *α* determined by particle tracking micro-rheology showed a strong linear correlation with CFU, PQS and HHQ (Fig. 3) with the absolute values of the Pearson’s correlation coefficients being greater than 0.90 in all cases. Consequently, the grade of infection of this particular model can be accurately estimated by determining the micro-rheological properties of lung homogenates. The MPT analysis revealed the different response to infection of each mouse. Even though all animals received the same inoculum of *P. aeruginosa*, the lung homogenates of mice with the lowest bacterial load (i.e. lowest CFU) displayed mean *α values* higher than 0.9 and the tracer particles appeared to be diffusing through a viscous fluid. Conversely, in those mice with the highest bacterial load (i.e. highest CFU), the tracer particles were almost exclusively immobilized by a biomolecular network with a pore size of approximately 200 nm. The strict pore size might have implications for nanoparticle-mediated drug delivery and may also limit the migration of immune cells in the setting of severe pneumonia.

The samples analyzed in this experiment represent tissue homogenates obtained from two independent experimental sessions with 4 or 8 mice in each session, respectively. Within each replicate, there is a similarly strong correlation between signaling molecules, CFU and logMSD/α (r > 0.87, Fig. S4 and a Table S3), showing reproducibility of our results.

These findings demonstrate our hypothesis that the mechanical properties of lungs from infected mice serve as reliable marker to assess the status of an infection with *P. aeruginosa*. Several factors may account for the overall increase of the viscosity in the setting of bacterial infections aside from the alginate overproduced by mucoid *P. aeruginosa* strains. For instance, high bacterial content, mucus hypersecretion, increased mucin cross-linking, and the development of a parallel network of DNA debris and cytoskeleton proteins from apoptotic neutrophils significantly increase the viscosity of CF sputum^24–27^. Our MPT investigations and those of others have shown that a significant fraction of 200 nm PEGylated particles (35–90%) display a diffusive behavior through pulmonary mucus and CF sputum^15,19,28^. It is noteworthy that in 3 out of 12 samples of this work almost 100% of the PEGylated tracer particles were immobilized by a biomacromolecular network. In vitro, *P. aeruginosa* biofilms show rheologically inhomogeneous structures at the microscale derived from the adhesion to the growth substrate, the arrangement of individual bacteria, and the formation of larger, multibacterial floc-like structures^29^. Moreover Gloag et al*.* have recently shown that *P. aeruginosa* mucoid biofilms progress to an elastic-solid behavior over time, proposing the viscoelasticity as a virulence factor of biofilms^30^. However, here we did not exclusively analyze the pulmonary lining fluid or the bacterial biofilms alone but the whole lung content, including lung tissue, after homogenization. Nevertheless, considering that particles dispersed in lung homogenates from animals with a mild infection showed a diffusive behavior, we hypothesize that the changes in the micro-rheological properties of the lung homogenates are principally associated with the proliferation of mucoid biofilms of the *P. aeruginosa* NH57388A alginate-hypersecreting strain.

## Conclusion

This study initially aimed to establish a murine sub-chronic lung infection model with mucoid *P. aeruginosa NH57388A*. Not only chronic but also acute murine lung infection models are difficult to establish and maintain due to high variances of survival and biomarkers within the infected population as well as between experiments. In the present study an alginate based murine lung infection model in a prolonged acute setting was successfully used. Although the biomarkers HHQ, PQS and CFU showed high variance, their positive correlation was very strong as was hypothesized. To the best of our knowledge, only weak to moderate positive correlations have been demonstrated before. Additionally, in the present study micro-rheological properties of lung homogenates were analyzed and shown to strongly correlate with HHQ (or PQS) and CFU as well. Especially in the case of NCFB, clotting of the patient’s bronchi by infection-mediated mucus plugs is a major parameter affecting organ function and causing pulmonary exacerbations. Although it is not known whether the aforementioned correlation stems from alginate overproduction of the mucoid strain or mucus overproduction of the host, gaining access to this clinically relevant study endpoint is of high relevance for (pre-) clinical development of mucolytic and non-traditional anti-infective drugs (e.g. pathoblockers). The data provided herein shed light on the potential of lung homogenate viscosity determined via MPT as an alternative study endpoint for assessing the effectiveness of non-antibiotic therapies against *P. aeruginosa* infections in in vivo models with only small sample volumes required.

## Supplementary information

Supplementary Information 1.

Supplementary Video 1.

Supplementary Video 2.

Supplementary Video 3.
