# Supplementary material for: Micro-rheological properties of lung homogenates correlate with infection severity in a mouse model of Pseudomonas aeruginosa lung infection
Source: Sci Rep. 2020 Oct 5;10:16502. doi: 10.1038/s41598-020-73459-5 (PMC7536435; doi:10.1038/s41598-020-73459-5)
Supplement: Supplementary file 1 — Supplementary Information 1. [file 41598_2020_73459_MOESM1_ESM.pdf]

Supplementary Material

Micro-rheological properties of lung homogenates correlate with infection severity in a mouse model of *Pseudomonas aeruginosa* lung infection

Xabier Murgia[1,6,7], Andreas M. Kany[2,3,6], Christian Herr[4], Duy-Khiet Ho[1], Chiara De Rossi[1], Robert Bals[4], Claus-Michael Lehr[1,5], Anna K.H. Hirsch[2,3,5], Rolf W. Hartmann[2,3,5], Martin Empting[2,3,5*], Teresa Röhrig[2,3,8*]

[*]Correspondence: martin.empting@helmholtz-hips.de (M.E.), teresa.roehrig@googlemail.com (T.R.)

[1] Department of Drug Delivery (DDEL), Helmholtz-Institute for Pharmaceutical Research Saarland (HIPS)-Helmholtz Centre for Infection Research (HZI), Helmholtz Institute for Pharmaceutical Research Saarland, Campus E8.1, 66123 Saarbrücken, Germany

[2] Department of Drug Design and Optimization (DDOP), Helmholtz-Institute for Pharmaceutical Research Saarland (HIPS)-Helmholtz Centre for Infection Research (HZI), Campus E8.1, 66123 Saarbrücken, Germany

[3] German Centre for Infection Research (DZIF), Partner Site Hannover-Braunschweig, Saarbrücken, Germany

[4] Department of Internal Medicine V – Pulmonology, Allergology, Critical Care Medicine, Saarland University Hospital, 66421 Homburg, Germany

[5] Department of Pharmacy, Saarland University, Campus E8.1, 66123 Saarbrücken, Germany

[6] These authors contributed equally

[7] Current address: Kusudama Therapeutics, Parque Científico y Tecnológico de Gipuzkoa, 20014 Donostia-San Sebastián

[8] Lead contact

Supplemental Figures

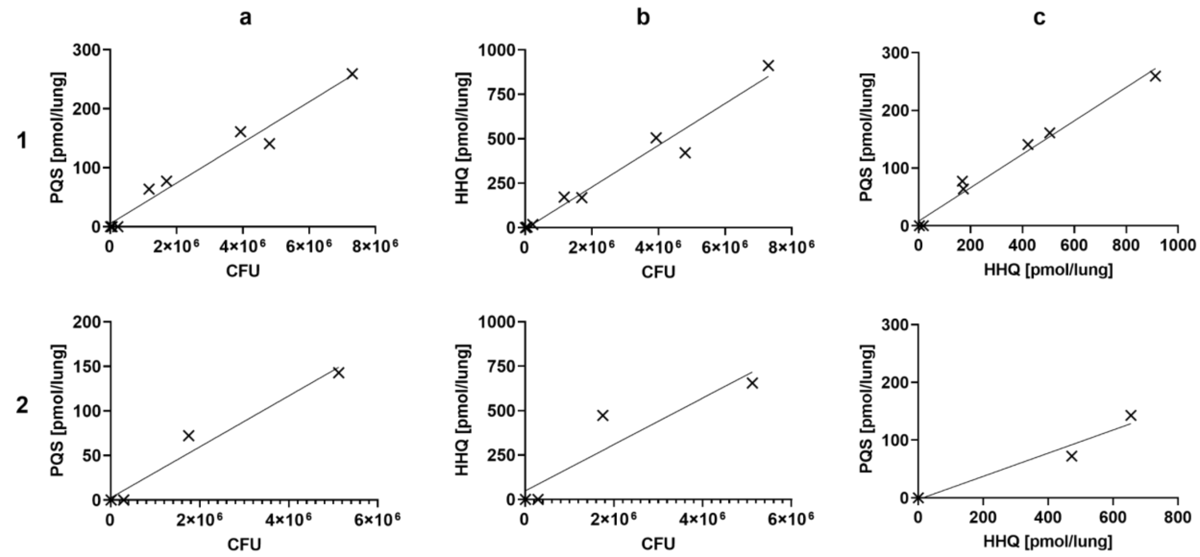

Figure S1. Related to Figure 1; Scatterplot and linear regression of the quorum sensing signaling molecule (a) PQS (2-heptyl-3-hydroxy-4(1H)-quinolone) *vs* corresponding colony forming units (CFU) detected in murine *Pseudomonas aeruginosa* NH57388A lung infection samples, (b) HHQ (2-heptyl-4-quinolone) *vs* CFU and (c) PQS *vs* HHQ, generated in experiment 1 and 2, respectively.

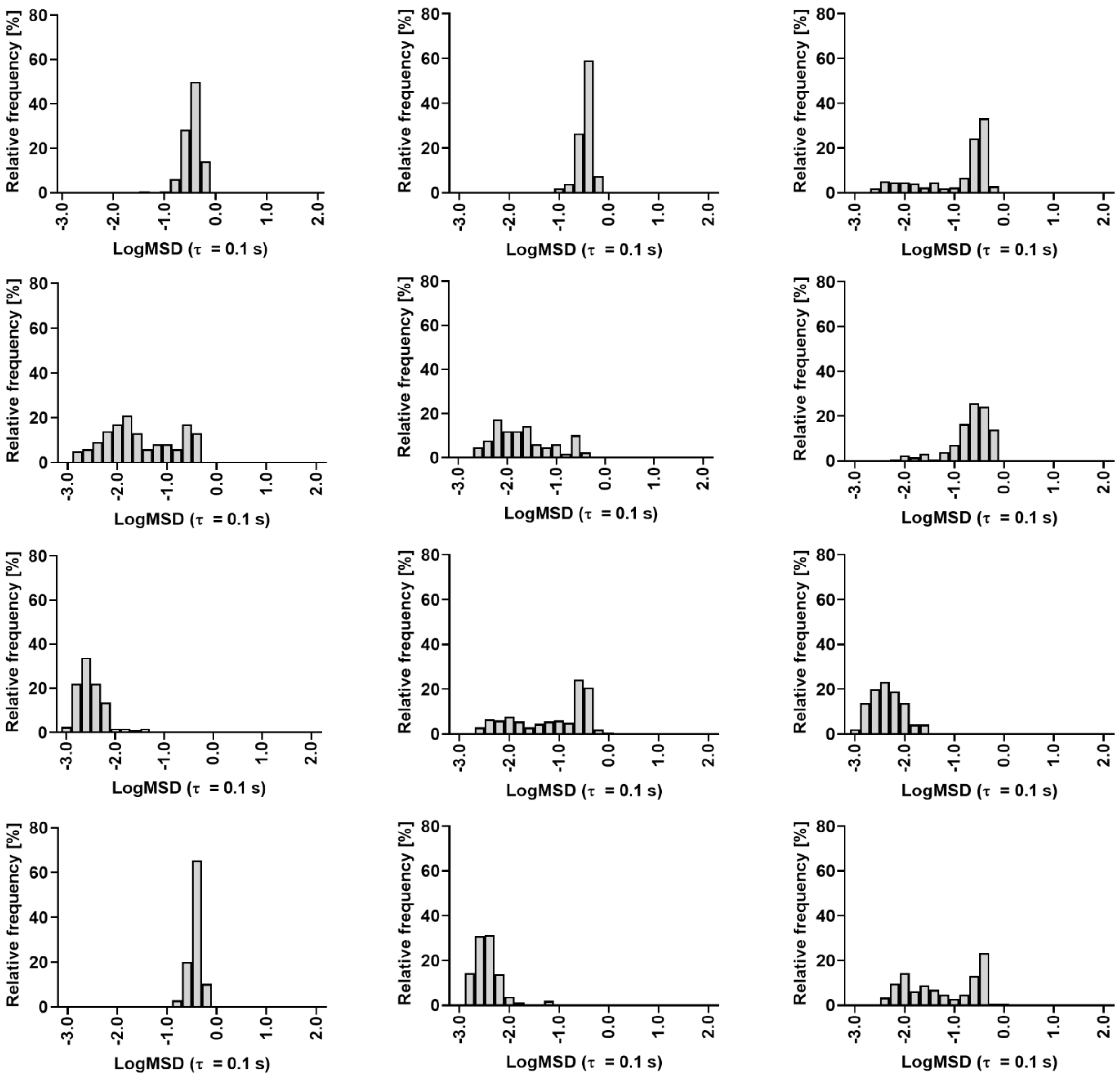

Figure S2. Related to Figure 2b; Logarithmic mean squared displacement (MSD) distribution at τ=0.1 s of fluorescent tracer particles in murine *Pseudomonas aeruginosa* NH57388A lung infection samples of experiment 1 and 2 determined *via* multiple particle tracking.

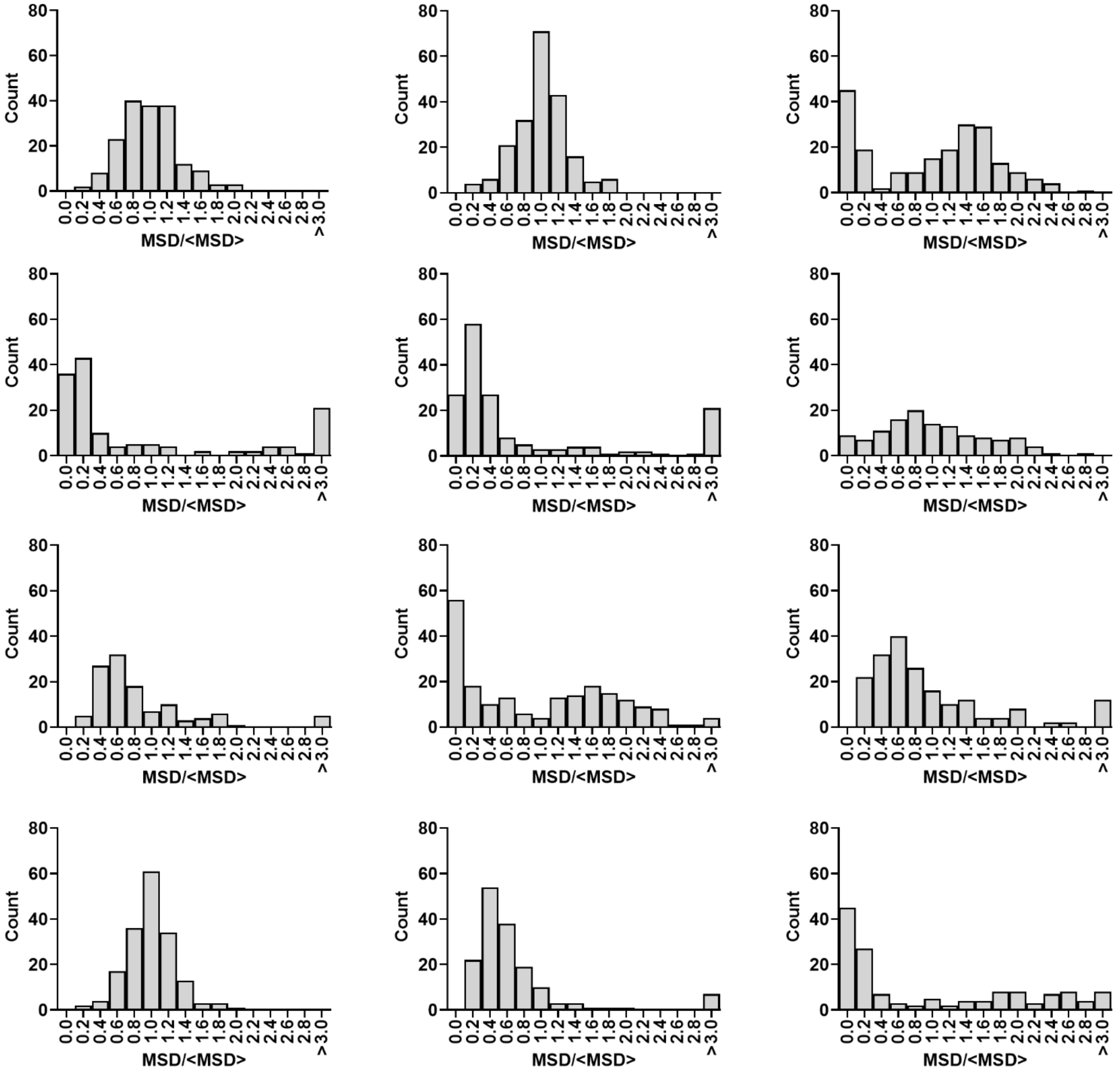

Figure S3. Related to Figure 2c. Representative mean squared displacement (MSD) distributions at τ=0.1 s normalized by the ensemble-average MSD (<MSD>) at τ=0.1 s.

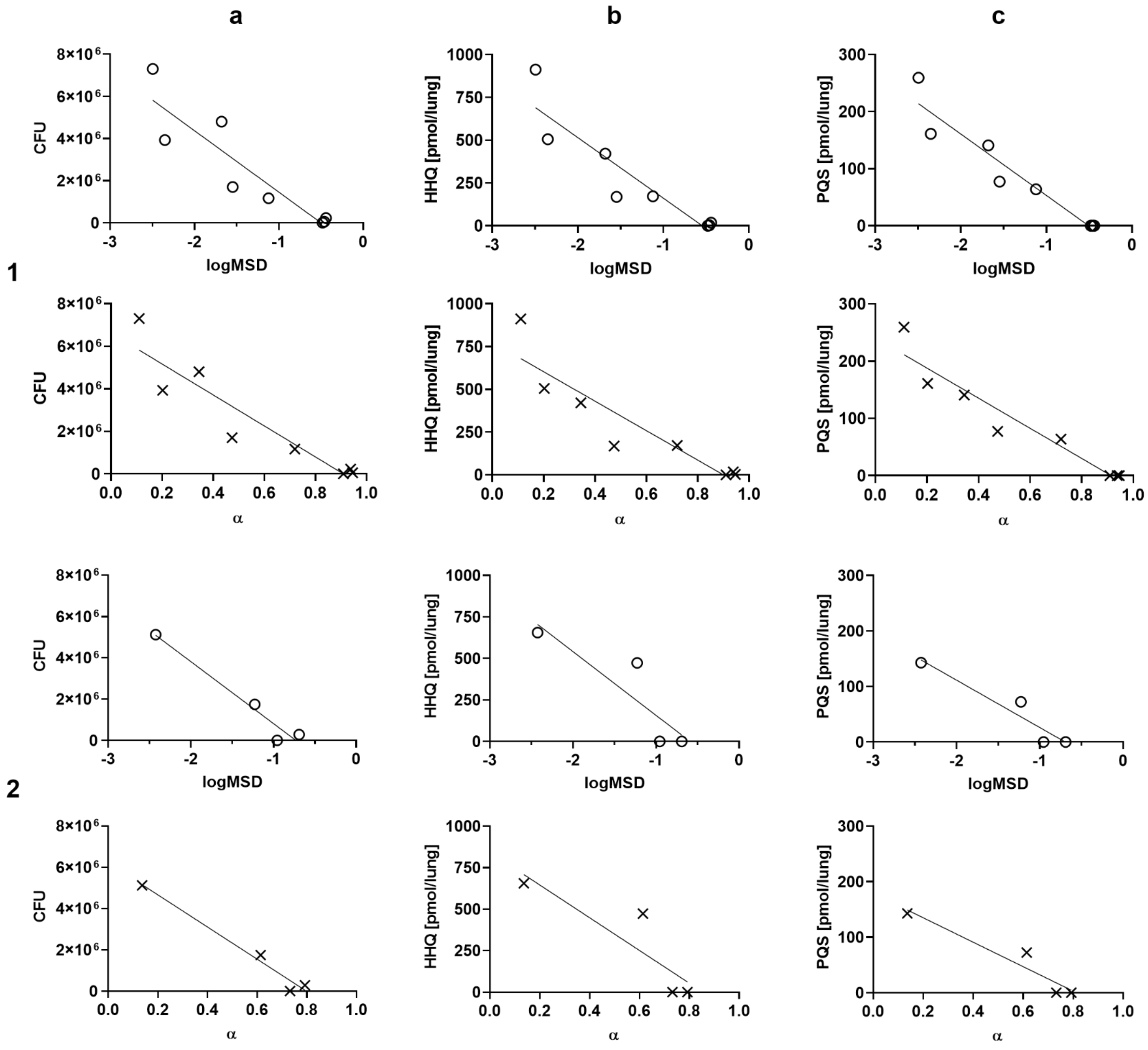

Figure S4. Related to Figure 3; Scatterplot and linear regression of (a) colony forming units (CFU), HHQ (b) and PQS (c) *vs* logMSD (o) and exponential coefficient α (x), as detected in murine *Pseudomonas aeruginosa* NH57388A lung infection samples of experiment 1 and 2, respectively.

Supplemental Tables

Table S1: Compound dependent reaction monitoring parameters in positive ionization mode and retention times of quorum sensing biomolecules PQS (2-heptyl-3-hydroxy-4(1H)-quinolone), HHQ (2-heptyl-4-quinolone), HQNO (N-oxo-2-heptyl-4-hydroxyquinoline) and stable isotope labeled standards.

	**precursor ion m/z**	**fragment ion m/z**	**collision energy (V)**	**tube lens offset (V)**	**retention time (min)**
**PQS**	260.048	145.958	44	110	2.4
		174.927	30	110	
**HHQ**	244.050	158.944	31	100	2.3
		171.943	33	100	
**HQNO**	260.036	158.908	28	110	3.0
**d4PQS**	264.048	178.927	30	110	2.4
**d4HHQ**	248.081	162.965	32	100	2.3
		175.982	34	100	

Table S2: Pearson's R corresponding to Figures 1 and 3.

	**PQS [pmol/lung]**	**HHQ [pmol/lung]**	**logMSD**	**α**
**CFU**	0.9769	0.9523	0.9201	0.9356
**HHQ [pmol/lung]**	0.9548		0.9080	0.9053
**PQS [pmol/lung]**			0.9344	0.9342

Table S3: Pearson’s R corresponding to Figures S1 and S4.

**Experiment 1**				
**Pearson‘s R**	**PQS [pmol/lung]**	**HHQ [pmol/lung]**	**logMSD**	**α**
**CFU**	0.9828	0.9810	0.9093	0.9311
**HHQ [pmol/lung]**	0.9910		0.9080	0.9200
**PQS [pmol/lung]**			0.9587	0.9600
**Experiment 2**				
**Pearson‘s R**	**PQS [pmol/lung]**	**HHQ [pmol/lung]**	**logMSD**	**α**
**CFU**	0.9802	0.9189	0.9791	0.9873
**HHQ [pmol/lung]**	0.9781		0.8801	0.8784
**PQS [pmol/lung]**			0.9551	0.9571

Supplemental Videos

Video S1: Representative video of lung homogenate with low viscosity.

Video S2: Representative video of lung homogenate with high viscosity.

Video S3: Representative video of lung homogenate with intermediate viscosity.
